# Measurement of the Neutron Lifetime by Counting Trapped Protons

**DOI:** 10.6028/jres.110.048

**Published:** 2005-08-01

**Authors:** F. E. Wietfeldt, M. S. Dewey, D. M. Gilliam, J. S. Nico, X. Fei, W. M. Snow, G. L. Greene, J. Pauwels, R. Eykens, A. Lamberty, J. Van Gestel

**Affiliations:** Tulane University, New Orleans, LA 70118; National Institute of Standards and Technology, Gaithersburg, MD 20899; Indiana University, Bloomington, IN 47408; University of Tennessee/Oak Ridge National Laboratory, Knoxville, TN 37996; European Commission, Joint Research Centre, Institute for Reference Materials and Measurements, 2440 Geel, Belgium

**Keywords:** neutron lifetime, trapped protons

## Abstract

We measured the neutron decay lifetime by counting in-beam neutron decay recoil protons trapped in a quasi-Penning trap. The absolute neutron beam fluence was measured by capture in a thin ^6^LiF foil detector with known efficiency. The combination of these measurements gives the neutron lifetime: *τ*_n_ = (886.8 ± 1.2 ± 3.2) s, where the first (second) uncertainty is statistical (systematic) in nature. This is the most precise neutron lifetime determination to date using an in-beam method.

## 1. Introduction and Discussion

As the simplest semi-leptonic decay system, the free neutron plays a crucial role in understanding the physics of the weak interaction and testing the validity of the Standard Model. The current experimental uncertainty in the neutron lifetime dominates the uncertainty in calculating the primordial helium abundance of the universe with Big-Bang nucleosynthesis [1]. Currently, there are six experiments [2–7] that contribute to the neutron lifetime world average of *τ*_n_ = (885.7 ± 0.8) s [8]. In the four most precise measurements, ultra cold neutrons were confined in a bottle; the decay lifetime is determined by counting the neutrons that remain after some elapsed time, with a correction for competing neutron loss mechanisms. The other two (in-beam) experiments [2, 6] measured the absolute specific activity of a beam of cold neutrons by counting decay protons. Given the very different systematic problems that the two classes of experiments encounter, a more precise measurement of the lifetime using the in-beam technique not only reduces the overall uncertainty of *τ*_n_ but also provides a strong check on the robustness of the central value.

**Fig. 1:**
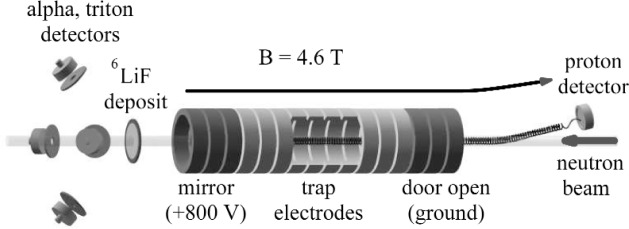
Schematic representation of the experimental method (not to scale).

We measured the neutron lifetime at the cold neutron beam NG6 at the National Institute of Standards and Technology (NIST) Center for Neutron Research, using the quasi-Penning trap method first proposed by Byrne et al. This method is described in detail in previous publications [9]. Figure 1 shows a sketch of the experimental configuration. A proton trap of length *L* intercepts the entire width of the neutron beam. Neutron decay is observed by trapping and counting decay protons within the trap with an efficiency *ε*_p_. The neutron beam is characterized by a velocity dependent fluence rate *I*(*v*). The rate *Ṅ*_p_ at which decay protons are detected is proportional to the mean number of neutrons inside the trap volume

$${\dot{N}}_{p}=\frac{\varepsilon_{p}L}{\tau_{n}}\int_{A}daI(v)\frac{1}{v},$$ (1)

where *A* is the beam cross sectional area. After leaving the trap, the neutron beam passes through a thin foil of ^6^LiF. The probability for absorbing a neutron in the foil through the ^6^Li(*n*, *t*)^4^He reaction is inversely proportional to the neutron velocity *v*. The reaction products, alphas or tritons, are counted by a set of four silicon surface barrier detectors in a well-characterized geometry. We define the efficiency for the neutron detector, *ε*_o_, as the ratio of the reaction product rate to the neutron rate incident on the deposit for neutrons with thermal velocity *v*_o_ = 2200 m/s. The corresponding efficiency for neutrons of other velocities is *ε*_o_*v*_o_/*v*. Therefore, the net reaction product count rate *Ṅ*_α_ is

$${\dot{N}}_{\alpha }=\varepsilon_{0}\nu_{0}\int_{A}daI(v)\frac{1}{v}.$$ (2)

The integrals in Eq. (1) and Eq. (2) are identical; the velocity dependence of the neutron detector efficiency compensates for the fact that the faster neutrons in the beam spend less time in the decay volume. This cancellation is exact except for a correction due to the finite thickness of the ^6^LiF foil (+5.4 s), and we obtain the neutron lifetime *τ*_n_ from the experimental quantities *Ṅ*_α_/*Ṅ*_p_, *ε*_o_, *ε*_p_, and *L*.

The proton trap was composed of sixteen annular electrodes, each 18.6 mm long with inner diameter of 26.0 mm, cut from fused quartz and coated with a thin layer of gold. Adjacent segments were separated by 3 mm-thick insulating spacers of uncoated fused quartz. The dimensions of each electrode and spacer were measured to a precision of ±5 μm using a coordinate measuring machine at NIST. Changes in the dimension due to thermal contraction are below the 10^−4^ level for fused quartz. The trap resided in a 4.6 T magnetic field, and the vacuum in the trap was maintained below 10^−9^ mbar.

**Fig. 2:**
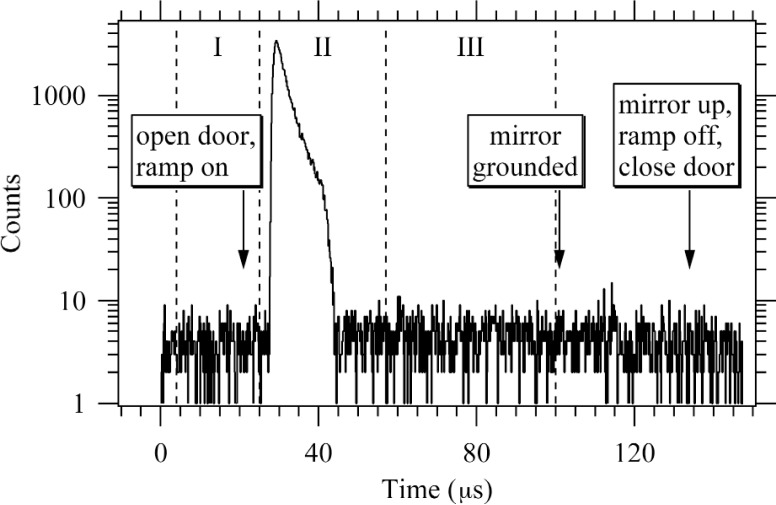
A typical plot of proton detection time after gating on the detector (*t* = 0). Regions I and III are used to subtract background from the peak region II. After correcting for deadtime in the time-to-digital converter, the resulting peak area gives the proton rate *Ṅ*_p_.

In trapping mode, the three upstream electrodes (the “door”) were held at +800 V, and a variable number of adjacent electrodes (the “trap”) were held at ground potential. The downstream three adjacent electrodes (the “mirror”) were held at +800 V. We varied the trap length from 3 to 10 grounded electrodes. When a neutron decayed inside the trap, the decay proton was trapped radially by the magnetic field and axially by the electrostatic potential in the door and mirror. After some trapping period, typically 10 ms, the trapped protons were counted. In counting mode, the door electrodes were lowered to ground potential, and a small ramped potential was applied to the trap electrodes to assist slower protons out the door. The protons were then guided by a 9.5° bend in the magnetic field to the proton detector held at a high negative potential (−27.5 kV to −32.5 kV). After the door was open for 76 μs, a time sufficient to allow all protons to exit the trap, the mirror was also lowered to ground potential. This prevented negatively charged particles, which may contribute to trap instability, from accumulating in any portion of the trap. That state was maintained for 33 μs, after which the door and mirror electrodes were raised again to +800 V, reverting to the trapping mode, and another trapping cycle began. Since the detector needed to be enabled only during extraction, the counting background was reduced by the ratio of the trapping time to the extraction time (typically a factor of 125). Figure 2 shows a plot of proton detection time for a typical run.

Protons born in the grounded electrode region inside the trap were trapped with 100 % efficiency. However protons that were born near the door and mirror (the “end regions”), where the electrostatic potential is elevated, were not all trapped. A proton born in the end region was trapped if its initial (at birth) sum of electrostatic potential energy and axial kinetic energy was less than the maximum end potential. This complication caused the effective length *L* of the trap to be difficult to determine precisely. To avoid this complication we varied the trap length. The shape of the electrostatic potential near the door and mirror was the same for all traps with 3–10 grounded electrodes, so the effective length of the end regions, while unknown, was to good approximation constant. The length of the trap can then be written *L* = *nl* + *L*_end_, where *n* is the number of grounded electrodes and *l* is the physical length of one electrode plus an adjacent spacer. *L*_end_ is an *effective* length of the two end regions; it is proportional to the physical length of the end regions *and* the probability that protons born there will be trapped. From Eqs. (1) and (2) we see that the ratio of proton counting rate to alpha counting rate is then

$$\frac{{\dot{N}}_{p}}{{\dot{N}}_{á}}=\tau_{n}^{-1}\left(\frac{\varepsilon_{p}}{\varepsilon_{0}\nu_{o}}\right)\left(nl+L_{\text{end}}\right).$$ (3)

**Fig. 3:**
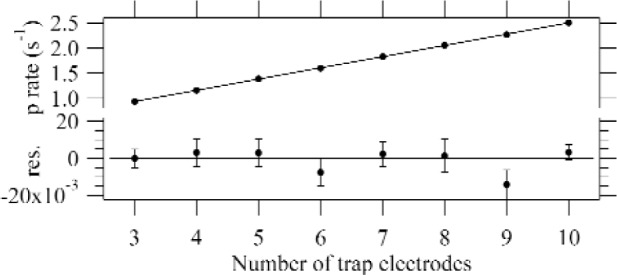
Typical raw proton count rate *Ṅ*_p_ vs trap length data, fit to a straight line (top), and residuals (bottom). These data have not yet been corrected for nonlinearities.

We fit *Ṅ*_p_ *Ṅ*_α_/as a function of *n* to a straight line and determine *τ*_n_ from the slope, so there is no need to know the value of *L*_end_, provided that it was the same for all trap lengths. Figure 3 shows raw data from a typical run, proton count rate vs trap length *n*.

Because of the symmetry in the Penning trap’s design, *L*_end_ was approximately equal for all trap lengths that we used. However there were three trap-length-dependent effects that broke the symmetry: the gradient in the axial magnetic field (the dominant effect), the divergence of the neutron beam (a minor effect), and the slight variation in geometry of the individual electrodes (a very small effect). Each of these caused *L*_end_ to vary slightly with trap length. A detailed Monte Carlo simulation of the experiment, based on the measured and calculated magnetic and electric field inside the trap, was developed in order to correct for these trap nonlinearities. It gave a trap-length dependent correction that lowered the lifetime by 5.3 s.

A variety of surface barrier (SB) and passivated ion-implanted planar silicon (PIPS) detectors were used to count the protons. The proton detectors were large enough so that all protons produced by neutron decay in the collimated beam, defined by C1 and C2, would strike the 19.7-mm diameter active region after the trap was opened. The detector was optically aligned to the magnetic field axis, and the alignment was verified by scanning with a low energy electron source at the trap’s center and with actual neutron decay protons. When a proton hit the active region of the detector, the efficiency for proton detection was less than 100 % due to proton backscattering—a proton can backscatter without depositing enough energy in the active region to be counted above threshold. This is complicated by the fact that an uncounted, backscattered proton has some probability to be reflected by the electrostatic acceleration field back into the detector and counted.

**Fig. 4:**
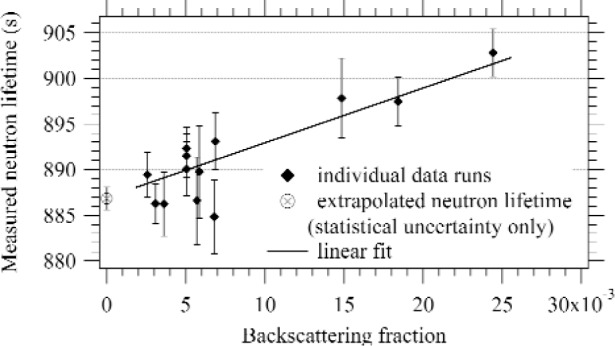
A linear fit of the measured neutron lifetime at varying values of the detector backscattering fraction. The extrapolation to zero backscattering gives the free neutron lifetime.

To determine the proton detection efficiency, we ran the experiment with a variety of detectors with different dead layer thicknesses and different acceleration potentials. The fraction of protons that backscatter were calculated using the SRIM 2003 Monte Carlo program [10]. We made an extrapolation of the measured neutron lifetime to zero backscatter fraction (see Fig. 4).

The neutron detector target was a thin (0.34 mm), 50-mm-diameter single crystal wafer of silicon coated with a 38 mm diameter deposit of ^6^LiF, fabricated at the Institute for Reference Materials and Measurements (IRMM) in Geel, Belgium. The manufacture of deposits and characterization of the ^6^LiF areal density were exhaustively detailed in measurements performed over several years [11]. The average areal density was *ρ* = (39.30 ± 0.10) μg/cm^2^. The α particles and tritons produced by the ^6^Li(*n*, *t*)^4^He reactions were detected by four surface barrier detectors, each with a well-defined and carefully measured solid angle. The geometry was chosen to make the solid angle subtended by the detectors insensitive to first order in the source position. The parameter *ε*_0_ gives the ratio of detected alphas/tritons to incident thermal neutrons. It was calculated using

$$\varepsilon_{0}=\frac{\sigma_{0}}{4ð}\iint \Omega \left(x,y\right)\rho \left(x,y\right)\theta \left(x,y\right)dxdy,$$ (4)

where *σ*_0_ is the cross section at thermal (*v*_0_ = 2200 m/s) velocity, *Ω*(*x*, *y*) is the detector solid angle, *ρ*(*x*, *y*) is the areal mass density of the deposit, and *θ* (*x*, *y*) is the areal distribution of the neutron intensity on the target. The ^6^Li thermal cross section is (941.0 ± 1.3) b [12]. It is important to note that we take the ENDF/B-6 1 *σ* uncertainty from the evaluation, *not* the expanded uncertainty, to be the most appropriate for use with this precision experiment. The neutron detector solid angle was measured in two independent ways: mechanical contact metrology and calibration with ^239^Pu alpha source of known absolute activity. These two methods agreed to within 0.1 %.

Proton and neutron counting data were collected for 13 run series, each with a different proton detector and acceleration potential. The corrected value of the neutron lifetime for each series was calculated and plotted vs backscattering fraction, as shown in Fig. 4. A linear extrapolation to zero backscattering gave a result of *τ*_n_ = (886.8 ± 1.2[stat] ± 3.2[sys]) s. The summary of corrections and uncertainties is shown in Table 1. Our result will be improved by an independent absolute calibration of the neutron counter, which would significantly reduce the two largest systematic uncertainties, in the ^6^LiF foil density and ^6^Li cross section. A cryogenic neutron radiometer capable of such a calibration at the 0.1 % level has recently been demonstrated [13], and we are pursuing this method further.

## Figures and Tables

**Table 1:** Summary of systematic corrections and uncertainties (in seconds) for the measured neutron lifetime

Source	Correction	Uncertainty
^6^LiF foil areal density		2.2
^6^Li cross section		1.2
Alpha detector solid angle		1.0
Solid angle correction for beam distribution	+1.5	0.1
LiF target thickness	+5.4	0.8
^6^LiF distribution in target	−1.7	0.1
Neutron losses in Si wafer	+1.3	0.5
Neutron beam halo	−1.0	1.0
Trap nonlinearity (Monte Carlo)	−5.3	0.8
Proton backscatter calc.		0.4

Proton counting statistics		1.2
Neutron counting statistics		0.1

Total	+0.2	3.4
